# A fully digital approach for MR compatible Time-of-Flight PET techniques

**DOI:** 10.1186/2197-7364-1-S1-A9

**Published:** 2014-07-29

**Authors:** Paul Lecoq

**Affiliations:** CERN, Meyrin, Switzerland

Time-of-Flight PET (TOFPET) is likely to bring a significant improvement in PET image quality that could have a high impact in PET/MR acquisition and image reconstruction procedures.

Silicon photomultipliers (SiPM) are known to be compatible with high magnetic fields. However, commercial SiPMs do not take advantage of their digital nature and are working in an analog mode, which make them sensitive to noisy environments. In order to be immune to the hostile environment of an MRI system we propose a fully digital approach, based on the Multidigital SiPM (MD-SiPM) we have developed in the frame of the FP7 funded EndoTOFPET-US project.

In parallel we propose to exploit transient phenomena in scintillators generating a few hundred photons in the picosecond range. We will review the different processes at work and evaluate if some of the transient phenomena taking place during the fast thermalization phase of hot electron-hole pairs produced by the conversion of the 511 KeV gamma rays can be exploited to extract a sub-100ps time tag.

Simulation results show how a multidigital approach makes the readout system much more robust and immune to a noisy environment. Characterization data and performance evaluation of our MD-SiPM will be shown as well as first unpublished results on the potential of exploiting transient phenomena in scintillators.

The potential of Time-of-Flight techniques in PET/MRI is presented. The merit of fully digital photon counting methods is discussed. The possibility to exploit ultrafast transient phenomena in specifically optimized scintillators, which can generate up to a few hundreds of photons in less than 10 ps, opens the way to coincidence timing resolution of better than 100ps at a system level, with a high potential for improving the image mapping and overall image quality of a combined PET/MRI machine.

